# A qualitative assessment of Ukraine’s trauma system during the Russian conflict: experiences of volunteer healthcare providers

**DOI:** 10.1186/s13031-024-00570-z

**Published:** 2024-01-25

**Authors:** Lynn Lieberman Lawry, Jessica Korona-Bailey, Luke Juman, Miranda Janvrin, Valentina Donici, Iurii Kychyn, John Maddox, Tracey Perez Koehlmoos

**Affiliations:** 1 Preventive Medicine and Biostatistics Department, Uniformed Services University, 4301, Jones Bridge Rd, Bethesda, MD 20814-4799 USA; 2 Henry M. Jackson Foundation for the Advancement of Military Medicine, Inc., 6720A Rockledge Drive, Bethesda, MD 20817 USA; 3 Bogomolets National Medical University, 13, T. Shevchenko Blvd., Kyiv, 01601 Ukraine

**Keywords:** Ukraine, Trauma systems, Emergency medical systems, Conflict health

## Abstract

**Background:**

The Russian Federation’s invasion of Ukraine is characterized by indiscriminate attacks on civilian infrastructure, including hospitals and clinics that have devastated the Ukrainian health system putting trauma care at risk. International healthcare providers responded to the need for help with the increasing numbers of trauma patients. We aimed to describe their experiences during the conflict to explore the gaps in systems and care for trauma patients to refine the Global Trauma System Evaluation Tool (G-TSET) tool.

**Methods:**

We conducted qualitative key informant interviews of healthcare providers and business and logistics experts who volunteered since February 2022. Respondents were recruited using purposive snow-ball sampling. Semi-structured, in-depth interviews were conducted virtually from January-March 2023 using a modified version of the G-TSET as an interview guide. Interviews were transcribed verbatim and deductive thematic content analysis was conducted using NVivo.

**Findings:**

We interviewed a total of 26 returned volunteers. Ukraine’s trauma system is outdated for both administrative and trauma response practices. Communication between levels of the patient evacuation process was a recurrent concern which relied on handwritten notes. Patient care was impacted by limited equipment resources, such as ventilators, and improper infection control procedures. Prehospital care was described as highly variable in terms of quality, while others witnessed limited or no prehospital care. The inability to adequately move patients to higher levels of care affected the quality of care. Infection control was a key issue at the hospital level where handwashing was not common. Structured guidelines for trauma response were lacking and lead to a lack of standardization of care and for trauma. Although training was desired, patient loads from the conflict prohibited the ability to participate. Rehabilitation care was stated to be limited.

**Conclusion:**

Standardizing the trauma care system to include guidelines, better training, improved prehospital care and transportation, and supply of equipment will address the most critical gaps in the trauma system. Rehabilitation services will be necessary as the conflict continues into its second year.

**Supplementary Information:**

The online version contains supplementary material available at 10.1186/s13031-024-00570-z.

## Introduction

Ukraine’s health system is devastated from indiscriminate attacks on civilian infrastructure, including hospitals and clinics by the Russian invasion since the start of the conflict on 24 February 2022 [1, 2]. Prior to the conflict, the underfunded Ukrainian healthcare system provided universal health care (UHC) using the Soviet era Shemashenko model producing limited access and unequal resource distribution [3]. The Ukrainian healthcare system is undergoing a transformation based on new objectives set by the Ministry of Health (MoH) in 2014. The transformation includes changes to emergency medical care and improved training programs following a Western model [3–6]. 

Emergency medical services (EMS) are part of the Ukrainian public healthcare system and include both prehospital EMS and facility-based emergency care with medics and Emergency Medicine trained physicians across all oblasts [7]. Training of prehospital emergency medical service (EMS) paramedics (called *feldshers*) predates the conflict. Approximately 1000 emergency medicine (EM) residency trained physicians work in Ukraine [7]. EM physicians were initially pre-hospital based but transitioned to hospital based care with the establishment of a regionally based network of EM capable hospitals and a national central dispatch system to coordinate EMS response. [7, 8]. 

The Ukrainian healthcare system is destabilized by the ongoing loss of personnel and the destruction of critical systems and infrastructure [1]. Loss of infrastructure and personnel is compounding the challenges facing the Ukrainian healthcare system [1]. As of November 2023, there were 1170 attacks that impacted health facilities and 111 healthcare providers killed and 189 injured [1]. Improving the delivery of trauma care to all casualties of war is increasingly difficult due to the destruction of equipment, ambulances, communications systems, in the setting of unreliable logistical support [1]. Trauma systems are comprised of coordinated functions, institutions, and personnel beginning with prevention and progressing through prehospital care, hospital care, and rehabilitation care to reintegration and survivorship [9]. In low- and middle-income countries (LMICs), and especially middle-income countries like Ukraine, the development of a trauma system is shown to progressively reduce traumatic injury mortality as the system matures [9]. Most Ukrainian healthcare providers do not have experience or training in a coordinated system of care approach to trauma [2, 10, 11]. Improving and maturing a coordinated trauma system approach to the overwhelming prehospital, hospital and rehabilitation care needs of the current conflict will bolster the Ukrainian healthcare system and ultimately improve mortality of those injured in the ongoing conflict [1–3, 12, 13].

Other than lessons learned publications that do not directly report qualitative data from volunteers, this is the first qualitative study of the experiences of international healthcare providers who volunteered alongside trauma specialists in Ukraine during the conflict [14, 15]. We aimed to describe their experiences during the conflict to explore the gaps in systems and care for trauma patients to modify the Global Trauma System Evaluation Tool (G-TSET) tool for further research.

## Methods

### Study design and respondents

We conducted semi-structed, in-depth key informant interviews (KIIs) of American and Canadian volunteers in a healthcare or healthcare adjacent capacity in Ukraine since February of 2022 to obtain detailed descriptions of experiences related to Ukraine’s trauma care system. Respondents had returned to their home counties.

Respondents were included if were healthcare or healthcare adjacent personnel who returned from and volunteered in Ukraine after 24 February 2022. Individuals who did not volunteer in a healthcare or health care adjacent position in Ukraine since 24 February 2022 or people currently providing services in Ukraine were excluded. Respondents were recruited using purposive and snow-ball sampling. We adapted the Global Trauma System Evaluation Tool (G-TSET) developed by military and civilian trauma specialists as an assessment tool for use in low- and middle-income countries [16]. The tool evaluates trauma systems by assessing the functional capacity of domains including leadership and organization, prevention of injuries, access to injury care, initial injury care, acute injury care, rehabilitation, and education, research, and quality improvement [16] (Additional File 1).

## Procedures

Volunteers were recruited via email from a list generated by non-governmental organizations (NGOs) who sent volunteers to augment trauma services throughout Ukraine. Virtual interviews were conducted using the Google Meets platform from 10 January – 6 March 2023. Two researchers conducted interviews in English: one guiding the discussion using the interview guide, and the second taking digital notes. Probes were used to gather as much detail as possible along G-TSET domains. The sample size was determined by saturation which was reached at 26 interviews. Confidentiality was assured by using a numerical code for each interview to deidentify transcripts.

## Data analysis

Deductive thematic content analysis was used to identify patterns or themes in the data and was guided by the assessment objectives and research questions using NVivo and open coding techniques [17]. We used the G-TSET tool as our framework to identify patterns that we expected to see in the data [16]. Any new patterns were identified and recorded through open coding where themes are identified as they are found in the data. NVivo was used to organize the data and pull out themes identified. The research team then manually summarized, categorized, and compared interview data and NVivo results to identify common themes from transcribed documents including expected themes from the G-TSET tool and any new themes. Resulting themes describe study respondents’ impressions of the trauma system in Ukraine (Table 1). Research team members selected and agreed upon illustrative quotes for each prevailing theme identified to limit any biases, subjectivity, assumptions, and experiences that may shape the research process and outcomes.

**Table 1:** Themes identified through deductive analysis of the modified G-TSET tool

Impressions and Organization of the Trauma System
Organization Communication Prehospital care challenges Prehospital evacuation and transport Hospital injury care challenges Care protocols Equipment challenges
**Education and Training Opportunities and Needs**
Education standards Training resources Gaps in education Telemedicine
**Rehabilitation Gaps and Needs**
Rehabilitation process Rehabilitation needs

## Role of the funding source

The funder of the study had no role in study design, data collection, data analysis, data interpretation, or writing of the report. The authors had full access to all the data in the study and had final responsibility for the decision to submit for publication.

## Results

### Characteristics of respondents

Our sample included 26 individuals (7 females; 19 males) between the ages of 25 and 70 who volunteered between February 2022 and February 2023. One participant was Canadian, and the remaining, American. Respondent characteristics are described in Table 2. The overarching themes that emerged from the analysis regarding the trauma system included trauma system impressions and organization education and training opportunities and needs and rehabilitation gaps and needs. Sub-themes are in Table 1.

**Table 2:** Characteristics of participants

Characteristic	
**Age (years), mean (range)**	55.2 (41–70)
**Gender**	
Male, n (%)	16 (69.5)
Female, n (%)	7 (30.5)
**Time in Ukraine (weeks), mean (range)**	5.4 (1–24)
**Occupation**	
Nurse, n (%)	4 (17.4)
Nurse Practitioner, n (%)	2 (8.7)
Physician, n (%)	4 (17.4)
Surgeon, n (%)	8 (34.8)
Other, n (%)	5 (21.7)
**Region** ^**a**^	
North, n (%)	11 (26.2)
South, n (%)	3 (7.1)
East, n (%)	7 (16.7)
West, n (%)	21 (50)
**Type of Facility**	
Hospital	18 (81.9)
Walk-in clinic	1 (4.5)
Role 1 & Role 2	2 (9.1)
Field ambulances	1 (4.5)

^a^ Volunteers worked in more than one region

### Trauma system impressions and organization

#### Organization

Respondents expressed concerns about the maturity and coordination and organization of the trauma system currently implemented in Ukraine stating the war has added system wide stress. The trauma system was described as a *“Soviet System”*, that follows outdated principles in clinical and administrative practices. And although there is a desire to modernize the system, the cultural phenomenon of hierarchical power limits change. They described the trauma system as rudimentary and lacking clear governance and a system that resembled that of the United States (US) 20–30 years prior, even back to the 1950s for burn injuries, Compared with US, there was an under-utilization of the nursing profession, lack of emergency medicine as a specialty and although there are *feltchers* (paramedics), they lacked training and were not integrated into the trauma system. *“Ukraine’s trauma system is not fully matured and lacks overall structure; it is very dependent on individual institutions instead of a systems wide approach. It does not have the benefit of the latest evidence-based practice guidelines in its implementation.” (Nurse 3)*.

#### Communication

Respondents felt the top-heavy hierarchical culture limited adequate communication. *“They do not tend to “speak out” about issues to their bosses.” (Surgeon 3)* The variability in leadership where the civilian hospitals have a divide between older and younger surgeons created tension for making changes. Communication difficulties existed between the military and civilian hospitals; “*A lot of it is hidden by operational security issues.*” (Surgeon 6) Communication between levels of the patient evacuation process was a recurrent concern mentioned: *“Trains would turn up on a specific day, but [from other facilities] we had no prior knowledge of who or what was on board; that would have been useful.” (Surgeon 8).* Record keeping was a stated issue and consisted of mostly handwritten notes. When electronic records were used, it was only by physicians, with nurses either relying on handwritten notes or nothing at all. Respondents suggested this would not be likely to change soon due to cultural differences and a lack of resources: *“They have almost no resources for that. It’s not a prominent part of the medical culture there. They realize* [electronic patient data collection] *is useful but given the lack of infrastructure, there is very little that is taking place.”* (Surgeon 6).

#### Prehospital care

Prehospital care was described as highly variable in terms of quality, while others witnessed limited or no prehospital care. One participant who volunteered in a forward surgical team (FST) described needing permission from a role 2 surgeon that added bureaucracy to urgent medical care and was part of the post-Soviet style of care. One participant stated board-certified specialists were deployed to front lines and could not provide adequate care or were killed. There was no organized system set up for testing, storage, or transportation of blood products.

#### Prehospital evacuation and transport

The timely transfer of patients between facilities and evacuation was stated as a main challenge. The inability to adequately move patients to higher levels of care affected the quality of care. *“Forward of the forward surgical team was vehicle transportation. Not always a* [marked with a]* Red Cross, just vehicles that they happened to have forward, anything from sprinter vans to pickup trucks to Land Rovers.” (Other Occupation 1)*.

“*Medical patients were held too long before being transported; and some already had systemic infections/septic; amputations have not been done for prosthesis or were not done in a way that the prosthesis would fit.*” *(Nurse Practitioner 2)* Closer to the frontlines, patients were transferred by trains that took 2–3 days to get to their treatment destinations or they were held too long before evacuation to higher levels of care. Concern was stated about the lack of a coordinated MEDEVAC system.

#### Hospital injury care challenges

Respondents described challenges in providing patient care in the hospital setting *“There was no consistent communication for patient medical data,” and “no formal process,” (Nurse Practitioner 1)*, often leading to a loss of information and poor clinical handoff of patients. Participants cited concerns of infection control as a key issue and hazardous conditions where patients were cared for in a badly ventilated “gymnasium-like structure” where the windows were allowed to stay open, temperatures were high, and insects were not controlled leading to compromised basic wound care.

#### Care protocols

Structured guidelines for trauma response were lacking and led to a lack of standardization of care and for trauma and especially burn patients. Respondents described handwashing and infection control protocols as *“non-existent”*. There were concerns of differences for standards of care. *“[there is a] Different mindset on soft tissue management. They wait longer to fix extremity fractures. People stay in external fixators for months at a time.”*(Surgeon 2) One respondent stated *“Post DCR* [damage control resuscitation] *and post DCS* [damage control surgery], *critical care transport is needed, because there are no* [intensive care units] *at Role 1* [unit that providing first aid, immediate lifesaving measures, and triage]. *This is the piece to be pushed, for life saving procedures.” (Physician 3)* Pain management was not the norm and antibiotics were given freely in the hospital and easily attainable at local pharmacies without a prescription. The ease of obtaining antibiotics often led to an increasing and significant burden of antimicrobial resistance.

#### Equipment challenges

Respondents reported an inability to maintain the stock of consumables needed for complex equipment such as ventilators or drills. Other unavailable items included ventilators, blood products, first aid equipment, oxygen, warming blankets, radiology equipment, and tourniquets; all made more difficult by bureaucracy. *“*[The process] *to obtain these supplies was just so bureaucratic that it made it unavailable for procurement and distribution.”(Other Occupation 2)*.

### Education and training

#### Education standards

Respondents noted *feldshers*, once trained were effectively employing tactical combat casualty care (TCCC) for military casualties. Training was offered through many different NGOs, academic institutions, and the United Nations.

#### Training resources

Training resources for Ukrainian providers, included TCCC, ATLS, first aid, infection control and handwashing. There were also lecture series for physicians on topics including traumatic injuries and traumatic brain injuries. Respondents stated a need for enroute critical care training.

#### Gaps in education

Respondents felt Ukrainians were eager to engage in education efforts, *“there was a huge hunger for education,”* and, *“*[Ukrainian healthcare professionals] *will do anything to gain that knowledge.” (Nurse Practitioner 2)* Others stated there was resistance to education efforts from Ukrainian healthcare professionals, with the consensus among them being, *“we do not need your guidelines*.” *(Surgeon 3)* The lack of bandwidth for training added to patient loads from the conflict might explain some of the resistance. [Providers] *“didn’t have the bandwidth* [for education efforts] *with what they were dealing with trying to care for patients,” (Nurse 3)* concluding that, *“education was probably not feasible for them at the time.” (Nurse3)*.

#### Telemedicine

Those who observed telemedicine being used stated it was limited to the sharing of photos between surgeons near the frontlines and those at facilities further West. A few respondents cited language, technology, and cybersecurity concerns as factors preventing widespread use of telemedicine.

### Rehabilitation needs and gaps

#### Rehabilitation process

Respondents were unsure what kind of rehabilitation care patients received after leaving the facilities, *“my impression was a lot of the rehab burden would fall on the families.” (Surgeon 4)* A few other respondents more familiar with rehabilitation efforts reported *“*[Ukrainian providers] *were sending a lot of individuals seeking rehabilitative care to neighboring countries.” (Physician 1)*.

#### Rehabilitation needs

Rehabilitation care was described as limited or non-existent. However, some respondents noted that *“this is something that the Ukrainians are conscious of and actively trying to resolve,” (Other Occupation 2)* and that, *“there is a great desire to develop rehab centers.” (Physician 1)* Respondents stated there was a need for robust physical therapy and occupational therapy components.

## Discussion

Using the G-TSET tool to understand these volunteer’s unique experiences and observations illustrate current challenges the Ukrainian healthcare and trauma systems are facing, allowing further research and aid efforts to address challenges more effectively.

### Lack of an organizational trauma system structure

The volunteer’s concern regarding the organization of the trauma system and the added system wide stress from conflict is multi-factorial. The lack of a standardized and mature trauma system will result in higher mortality and morbidity [9]. Wartime destruction of Ukraine’s critical healthcare infrastructure and systems has destabilized the healthcare systems and prevented the changes needed to provide optimal care of traumatically injured casualties of conflict. Although there were updates to the healthcare system including emergency medical care and EMS prior to the conflict, these have stalled and will be hard to implement during active conflict [3, 4, 7]. 

According to respondents, the inability to adequately move patients to higher levels of care affected the quality of care, morbidity and mortality of trauma patients. The “golden hour” or the first 60 min after a traumatic injury is the time that a severely injured patient has the best chance for survival if definitive medical care is reached within this timeframe [18]. Transportation of the injured to a medical facility at the front lines within the “golden hour” is unlikely due to current constraints of the conflict. The evacuation times combined with the overwhelmed, disabled and/or destroyed medical facilities significantly impact mortality and morbidity of traumatic injury patients [18]. Utilizing aeromedical evacuation would greatly improve evacuation times but is not currently feasible due to contested airspace in the combat area [7]. The development of a coordinated national medical evacuation system would improve evacuation times and clinical communication. Developing and employing a trauma registry is a feasible and critical trauma system improvement which will serve as the information backbone for all systems wide process improvement. A trauma registry is fundamental to a responsive and dynamic trauma system. A trauma registry, lacking in Ukraine, collects information related to the injury event, injuries, care, and outcomes across the entire continuum of trauma care. These data allow for a systematic analysis to guide ongoing quality improvement, resource allocation, and policy change [19]. In collaboration with Ukrainian colleagues, on-going research using a modified G-TSET tool, is exploring what approaches, policies, standards of care, and guidelines are necessary to strengthen the trauma care system in Ukraine in addition to a trauma registry (Additional File 1). Organization of the trauma and EMS must be determined by policies enacted into law. Since the start of the war, several orders from the Ministry of Health addressed shortcomings observed by respondents including standardization of care using disaster medicine world standards. Ultimately, only Ukraine will determine how best to develop a trauma system to meet their needs and constraints [20, 21].

### Hospital care challenges

The volunteers listed several issues in care they felt did not meet accepted guidelines such as pain relief, damage control resuscitation (DCR), antibiotic overuse leading to antibiotic resistant infections. Care protocols, even since the start of the conflict, have been updated and formalized as MOH orders [14, 15, 20]. Specifically, DCR guidance, pre-hospital care, burn guidance and other care challenged mentioned in other lessons learned documents such as tourniquet conversion and triage [14, 15, 20] And a new law requires a prescription for antibiotics, however, occupied territories or territories with active conflict are exempt, leaving areas where there are high numbers of injury vulnerable to continued antibiotic overuse [22].

### Communication challenges

Respondents had concerns about handwritten and incomplete notes that did not follow the patient across different levels and locations of care. Although, in the near term a computerized system is ideal, this would require the MOH to build information technology infrastructure and create guidelines and laws that will take time. Social media and mobile apps readily used in Ukraine could be considered for pre-hospital care and remote follow-up of patients in addition to improving standardized paper forms. Electronic registries like the electronic Trauma Health Record (eTHR) already being used in LIMCs could also be considered provided that the digital signal does not create a potential target for attack [23, 24]. The Ukrainian eHealth system is mandatory for all primary health care physicians since 2020 with planned expansion to complete implementation by 2024. A trauma registry or trauma health record should be compatible with the eHealth system given that anticipated ongoing rehabilitation needs long term follow up will be a primary care physician responsibility [23, 24]. Use of the Ukrainian EHR is expected to continue to expand, with the goal of complete implementation in 2024 [23, 24]. However, respondents reported limited patient data collection and storage despite Ukraine’s efforts to expand the eHealth system [23, 24]. Credible cybersecurity concerns may also drive the use of handwritten notes despite ongoing security countermeasures such as removal of geolocation and personnel contact information from the record [25]. Measures have been taken to alleviate these concerns, including the removal of geographic data and doctor contact information from the EHR management system, and further investment in cybersecurity measures [25]. While telemedicine is being used in Ukraine among civilians for primary care, the role of telemedicine for trauma care in Ukraine was reported but significantly limited [26, 27]. The language barrier, technological infrastructure, and ongoing cybersecurity concerns will be important factors to consider when expanding telemedicine use in Ukraine. Further research to determine how telemedicine is being used to augment trauma care is ongoing.

### Equipment and logistical challenges

A new MOH order was enacted to address the notable equipment challenges and the bureaucratic system [28]. Donations from governments and NGOs will be necessary given the continued destruction of healthcare infrastructure. Changes to Ukrainian UHC will be needed to address the out-of-pocket expenditures incurred by trauma patients.

### Education and training

A focus on evidence-based guidelines and protocols decreases morbidity and mortality within a medical system [12]. During conflict, the majority (87%) of preventable deaths occur in pre-hospital settings from hemorrhage [29]. Point of injury care training must focus on TCCC skills for applying tourniquets, and hemostatic dressings, to stem blood loss [29]. Prolonged field care guidelines appropriate to the Ukraine trauma system will help inform trauma care given the inability to transfer patients in a timely fashion [12]. In addition to existing clinical practice guidelines, guidelines for the management of critically ill patients, infection control, and surgical management of trauma patients will strengthen the Ukrainian system [20, 21].

The current role of nursing in the Ukrainian system limits their potential as a member of the clinical care team. One noted gap in education and training offerings were courses that expand the role of nursing in Ukraine [30]. Many of our respondents mentioned that nursing care in Ukraine is much more limited than in the United States (US); most of the medical care is provided directly by doctors. This is consistent with findings from WHO regarding primary care nursing in Ukraine and consistent with other health systems that evolved from a Shemashenko model [7]. Course offerings, like the *feldsher* training, that empower paramedics to take on more of the patient care and allow doctors to focus on complex care, will increase overall access to care [7, 8]. 

### Rehabilitation

Rehabilitation services represent a critical opportunity for improving the Ukrainian medical systems response to the casualties of the war [31, 32]. Rehabilitation is rarely prioritized as part of conflict responses but is critical to the recovery of injured patients [32]. Prior to the conflict Ukraine recognized that rehabilitation is a key component of universal health coverage however, there is no national rehabilitation strategy acknowledging the critical role of rehabilitation in a trauma system [32]. International partners with rehabilitation experience will be helpful in scaling up rehabilitation care programs to address the long-term disability resulting from conflict associated injuries [33]. 

### Limitations

Respondent’s perspectives are limited to their location and the timeframe of February 2022-February 2023. Participants were in Western or Central Ukraine; thus, the study lacks perspectives of volunteers in the Eastern part of the country closer to the front and do not represent the views of Ukrainian healthcare providers. Volunteers were recruited to assist in the care of conflict-related trauma. This study does not represent views of non-conflict trauma systems. Furthermore, most of the respondents did not have emergency and/or development experience in LMICs and therefore had only a U.S. or Canadian frame of reference. Anecdotal reports would need to be corroborated with current field information, through upcoming on the ground research.

## Conclusion

This study explores the gaps and needs in the Ukrainian trauma system including prehospital, hospital and rehabilitation care, and education and training through the eyes of Western volunteers using the G-TSET tool. While this was a rapid test of a modified G-TSET tool, our team in collaboration with Ukrainian research partners is currently conducting a study of Ukrainian health providers with an expanded version of the tool to develop an understanding of the situation and inform current and future medical needs and support for Ukraine based on the views of Ukrainians.

### Electronic supplementary material

Below is the link to the electronic supplementary material.

Additional File 1

## Data Availability

Data that support these findings are curated by the study team and are not available for public distribution.

## Abbreviations

ATLS: Advanced Trauma Life Support
CMAT: Canadian Medical Assistant Teams
DCS: Damage Control Surgery
EHR: Electronic Health Record
EM: Emergency Medicine
EMS: Emergency Medical Service
eTHR: Electronic Trauma Health Record
GSMSG: Global Surgical and Medical Support Group
G-TSET: Global Trauma System Evaluation Tool
IRB: Institutional Review Board
KII: Key Informant Interview
LMIC: Low-or Middle-Income Country
MOH: Ministry of Health
NGO: Non-governmental Organization
TCCC: Tactical Combat Casualty Care
US: United States
UHC: Universal Health Care
WHO: World Health Organization
