# Supplementary material for: A qualitative assessment of Ukraine’s trauma system during the Russian conflict: experiences of volunteer healthcare providers
Source: Confl Health. 2024 Jan 25;18:10. doi: 10.1186/s13031-024-00570-z (PMC10809523; doi:10.1186/s13031-024-00570-z)
Supplement: Supplementary file 1 — Additional File 1 [file 13031_2024_570_MOESM1_ESM.pdf]

GLOBAL TRAUMA SYSTEM EVALUATION TOOL (G-TSET)

COMPONENTS OF THE SYSTEM:
1. Leadership and Organization
2. Prevention of Injury
3. Access to Injury care
4. Initial Injury Care (District Hospitals/Clinics)
5. Acute Injury Care (Surgical Care and Critical care)
6. Rehabilitation
7. Education, Research, and Quality Improvement

SCORE CRITERIA:	
Not known	0
No	1
Minimal	2
Limited	3
Substantial	4
Full	5

COMPONENT: Leadership and Organization (100)

LEAD AGENCY: LEAD AGENT: CONTACT INFO:

BENCHMARK	INDICATOR	SCORING SYSTEM	SCORE	COMMENTS
101 - Establish key leadership with overarching legal authority				
	101.1 - Political leadership established for system of care			
	101.2 - Military leadership established for system			
	101.3 - Medical leadership established for system			
	101.4 - Medical leadership established for individual hospitals			
	101.5 - Military leadership established for each medical unit (MILITARY ONLY)			
102 - Trauma System Resource Assessment has been completed and is regularly updated				
	102.1 - Medical components of system have been identified (pre-hospital/access, district hospitals, regional hospitals)			
	102.2 - Medical component evaluations have been performed (individual hospital evaluations completed and available for reference)			
	102.3 - Gap analysis for the system of care is performed regularly			
	102.4 - New initiatives introduced into system as a result of ongoing gap analysis			
	102.5 - Military medical Roles of Care established and medical units are trained and assigned to their respective roles of care (MILITARY ONLY)			
103 - Establish communication within the system leadership				
	103.1 - System wide key leader meeting held at least quarterly (political, military, and medical key leaders to discuss status of the system)			
	103.2 - Component key leader meetings held at least monthly (hospitals, medical units (MILITARY), pre-hospital, ICU, rehabilitation, etc)			
	103.3 Military key leaders meeting at least monthly (MILITARY)			
	103.4 - System Assessments, Identification of problems, Development of interventions, and Evaluation of interventions (AIDE process) is reviewed at meetings at every level for continuity			
104 - Establish medical control communication within the system				
	104.1 - Establish nationwide telephone number for trauma and emergency care access (or equivalent activation plan)			
	104.1 - Establish radio communication between roles of medical care and a centralized battlefield Trauma System Director, a centralized command facility, and the ability for bi-directional communication (MILITARY ONLY)			
	104.2 - Establish communication between medical field assets (vehicles and personnel) and medical facilities			
	104.3 - Establish communication between medical facilities			

	104.4 - Establish overall Trauma and Emergency Medical Director for command, control, and communication (patient movement decisions and mascal C3); this includes a centralized command post and bi-directional communication capability			
	104.5 - Establish communication plan with police, fire, military, other agencies that react to disasters or mass casualty incidents			
105 - Establish a mass casualty plan of action				
	105.1 - Establish Disaster and MASCAL medical command center plan			
	105.2 - Establish Disaster and MASCAL medical communication and control plan			
	105.3 - Ensure medical disaster and mascal plan is integrated into national/state/regional mascal leadership and plan			
106 - Leadership provides feedback to populace regarding benefits of trauma and emergency care system				
	106.1 - Political leadership reports to public at least yearly regarding status of trauma system and benefits/advances			
	106.2 - Military leadership educates service members regarding system of trauma care established for their benefit			
107 - Lead Agents of each component adopt trauma system standards of care at each level				
	107.1 - Public Health (political and medical) leadership establish standards of care for hospitals and other components of system			
	107.2 - Component leaders develop standards of care for pre-hospital provider care (EMT B, etc), hospital care (level of hospital responsibility for trauma care based on capability of that facility), transfer guidelines between facilities, triage guidelines for pre-hospital care, data collection and reporting standards			
108 - Leadership at all levels develops Trauma System/Center Plan and Vision for Future				
	108.1 - National development of trauma system plan and vision			
	108.2 - Regional/city development of trauma system plan and vision			
	108.3 - Military trauma system plan and vision for deployed care			
	108.4 - Trauma system plan is based on analysis of the trauma demographics and resource assessments			
	108.5 - The trauma system plan clearly describes the system design (including the components necessary to have an integrated and inclusive trauma system) and is used to guide system implementation and management			
	108.6 - There is within the trauma system plan congruence of the population demographics with system development and resource allocation priorities (MILITARY considerations, pediatrics, burn, etc)			
	108.7 - A written injury prevention plan is developed, coordinated with other health agencies, is data driven when able, and targets high yield and risk injuries			
109 - There exists a financial commitment to the trauma system from the political governing body of the nation/state				
	109.1 - Funding is allocated to assist with all components of the trauma system			
110 - Trauma System leadership makes policy decisions based on available injury and emergency care data for the nation/state/region				

	110.1 - Data is provided to the system leadership from population and/or hospital based sources			
	110.2 - Policy development incorporates data on injury and emergency care provided by population and hospital based registries			

COMPONENT: Prevention of Injuries (200)

LEAD AGENCY: Lead Agent: CONTACT INFO:

BENCHMARK	INDICATOR	SCORING SYSTEM	SCORE	COMMENTS
201 - Establish Lead Agency and Agent for Prevention				
	201.1 - Establish legal authority for lead agent for system injury and emergency prevention issues			
	201.2 - Lead Agency and agent is clearly designated			
	201.3 - Lead Agency/agent receives data from other components and trauma registries			
	201.4 - Lead Agency/agent has direct communication with trauma and emergency system leadership and attends system meetings at all levels			
	201.5 - There is a lead agent for battlefield and non-battle injury prevention initiatives and this agency/agent has a direct route to military command leaders for implementation and evaluation of initiatives (MILITARY ONLY)			
202 - An Injury Prevention Program is established for Nation/State/Region				
	202.1 - Received data is analyzed on a frequent, no less than quarterly basis, to identify prevention interventions			
	202.2 - Prevention agency has identified and implemented at least one high yield prevention intervention for the system			
	202.3 - Prevention agency seeks ongoing feedback from registries regarding effectiveness of prevention interventions			
	202.4 - Prevention agency seeks cooperation and collaboration with other medical agencies and with public health officials and community leadership as available			
	202.5 - Prevention agency integrates prevention into the national trauma system plan			
203 - Injury prevention programs are politically supported				
	203.1 - Injury prevention plans communicated to system leadership and to Nation/State/Region political leadership			
	203.2 - Injury prevention plans are supported by all levels of leadership			
	203.3 - Injury prevention plan collaboration exists between EMS, public health officials, and trauma system leaders			
	203.4 - Injury prevention agency/agents have direct access to military commanders on all levels to assist with implementation of efforts			
204 - Injury prevention program public awareness campaigns are conducted				
	204.1 - Prevention agency conducts programs to educate the public regarding prevention initiatives			
	204.2 - Prevention agency accepts direct feedback from public and political bodies regarding efforts			

	204.3 - Prevention agency develops partnerships with community groups to raise awareness of injury as a disease, prevention issues, and the need for trauma and emergency care systems			

COMPONENT: Access to Injury Care (300)

LEAD AGENCY: Lead Agent: CONTACT INFO:

BENCHMARK	INDICATOR	SCORING SYSTEM	SCORE	COMMENTS
301 - Establish Pre-hospital oversight				
	301.1 - Establish lead agent for EMS director			
	301.2 - Establish battlefield trauma care director (MILITARY ONLY)			
	301.3 - Establish a lead agency for pre-hospital oversight			
	301.4 - Establish an access communication method (like 911 phone number system)			
	301.5 - Establish an EMS control center with bidirectional communication			
	301.6 - Establish a battlefield medical evacuation communication plan and a medical control center (MILITARY ONLY)			
302 - Establish Pre-hospital trauma and emergency care plan				
	302.1 - Establish written policy for pre-hospital care in the Nation/State/Region			
	302.2 - Establish pre-hospital care at scene plan			
	302.3 - Establish pre-hospital provider standards of care. Regardless of type of pre-hospital provider used, lay person or trained medical personnel, standards of care should be established.			
	302.4 - If lay person care is intended in this phase of care, there should be a link to the leadership arm of the system in order to provide awareness and education to the population and to create laws enabling lay person responsibility and care.			
	302.5 - Battlefield care plan established with buddy aid standards and ground medical personnel standards of care (MILITARY ONLY)			
303 - Establish Pre-hospital Transport plan				
	303.1 - Establish method of transport to a trauma center. This may be private vehicle or medical vehicle depending on Nation/State/Region laws and policy.			
	303.2 - Establish medical evacuation plan for combat (MILITARY ONLY)			
	303.3 - Establish transport enroute treatment plans if needed.			
	303.4 - Establish triage criteria for transport decisions. If lay transport is used, ensure link to leadership section for education and awareness of the populace.			
	303.5 - Establish pre-hospital communication plan with EMS control and trauma centers if trained medical personnel and medical vehicles used			
304 - Establish pre-hospital personnel, supplies, and equipment				
	304.1 - Conduct assessment of Pre-hospital service organizations if applicable. Conduct evaluation for each agency and attach to survey. See PRE-HOSPITAL EVALUATION TOOL (based on WHO Matrix of essential knowledge, skills, equipment, and supplies for prehospital providers).			
	304.2 - Establish pre-hospital equipment and personnel standards.			
305 - Establish pre-hospital training standards for personnel				

	305.1 - Establish pre-hospital medical care training and evaluation standards.			

COMPONENT: Initial Injury Care (District and Regional Hospitals/clinics) (400)

LEAD AGENCY: Lead Agent: CONTACT INFO:

BENCHMARK	INDICATOR	SCORING SYSTEM	SCORE	COMMENTS
401 - Establish lead agent for Trauma Center (For facilities only providing initial stabilization care for trauma)				
	401.1 - Establish a lead, highest level of care, trauma center.			
	401.2 - Establish a lead trauma provider (physician, nurse, other trained trauma provider) for each trauma center			
	401.3 - Establish clearly defined role for each trauma center within the system with its designated capability for level of care based on resources, equipment, supply, and personnnel			
	401.4 - Facilities are included in the trauma system (and written into the trauma system plan) from the district hospital/clinic initial care and stabilization level to the best capability hospital center for the State/Nation/Region.			
	401.5 - Each medical facility lead agent has designated authority by the medical facility leadership.			
402 - Establish transfer agreements and understandings between trauma care facilities of the lowest to the highest capability to facilitate patient movement within the system to the appropriate level of care in a timely fashion.				
	402.1 - Transfer agreements between all levels of initial care are established			
	402.2 - Methods of transfer between all levels of initial care established			
	402.3 - Ensure all level of trauma facilities are integrated into disaster and mass casualty plans.			
403 - Establish Trauma facility data collection and use				
	403.1 - Each trauma facility collects data and contributes data to the system.			
	403.2 - Each trauma facility receives feedback based on evaluation of trauma data from the trauma system leadership.			
404 - All trauma facilities have evaluations completed for their initial trauma care areas.				
	404.1 - Complete assessment of the initial trauma and emergency care for each facility in the system (WHO Needs Assessment and Evaluation Form for Resource Limited Heatlh Care Facility and WHO Essential Emergency Equipment List and WHO Anesthesia evaluation)			

COMPONENT: Acute Injury Care (Surgical Care and Critical care) (500)

LEAD AGENCY:	Lead Agent:	CONTACT INFO:		
BENCHMARK	INDICATOR	SCORING SYSTEM	SCORE	COMMENTS
501 - Establish lead agent for Trauma Center (For facilities providing both initial trauma and emergency care but also referral/tertiary care or ongoing/complete trauma care)				

	501.1 - Establish a lead, highest level of care, trauma center. This trauma center should be capable of providing the full spectrum of trauma care services from initial care through rehabilitation. It should provide full leadership within the system for all trauma care centers under the overall trauma system leadership.			
	501.2 - Establish a lead trauma provider (physician, nurse, other trained trauma provider) for each trauma center			
	501.3 - Establish clearly defined role for each trauma center within the system with its designated capability for level of care based on resources, equipment, supply, and personnnel			
	501.4 - Facilities are included in the trauma system (and written into the trauma system plan) from the district hospital/clinic initial care and stabilization level to the best capability hospital center for the State/Nation/Region.			
	501.5 - Each medical facility lead agent has designated authority by the medical facility leadership.			
502 - Establish transfer agreements and understandings between trauma and emergency care facilities of the lowest to the highest capability to facilitate patient movement within the system to the appropriate level of care in a timely fashion.				
	402.1 - Transfer agreements between initial care centers to full service centers are established			
	402.2 - Methods of transfer between all levels of initial care established			
	402.3 - Ensure all level of trauma facilities are integrated into disaster and mass casualty plans.			
503 - Establish Trauma facility data collection and use				
	503.1 - Each trauma facility collects data and contributes data to the system.			
	503.2 - Each trauma facility receives feedback based on evaluation of trauma data from the trauma system leadership.			
504 - All full service trauma facilities have evaluations completed detailing their individual center capabilities.				
	404.1 - Complete assessment of each full service facility in the system (WHO Tool for Situational Analysis to Assess Emergency and Essential Surgical Care and WHO Anesthesia survey)			

COMPONENT: Rehabilitation (600)

LEAD AGENCY: Lead Agent: CONTACT INFO:

BENCHMARK	INDICATOR	SCORING SYSTEM	SCORE	COMMENTS
601 - Establish lead agent for rehabilitation for each trauma center				
	601.1 - Designate lead agent from rehabilitation			
	601.2 - Empower lead agent to participate in trauma system planning			
602 - Each full service trauma center has a rehabilitation program				
	602.1 - Establish inpatient rehabilitation program with physicians, physical therapy, occupational therapy			
	602.2 - Establish facilities at trauma center suitable for performing essential rehabilitation			

	602.3 - Establish outpatient rehabilitation program with physicians, physical therapy, occupational therapy			
	602.4 - Establish facilities for outpatient rehabilitation			
603 - Link rehabilitation services to return to productive work and quality of life				
	603.1 - Develop programs that have rehabilitation, prosthetics, ongoing physician care to empower patients to return to QOL and work			
	603.2 - Ensure rehabilitation efforts are linked with overall trauma plans and with prevention efforts within the system			

COMPONENT: Education, Research, and Quality Improvement (700)

LEAD AGENCY: Lead Agent: CONTACT INFO:

**BENCHMARK**	**INDICATOR**	**SCORING SYSTEM**	**SCORE**	**COMMENTS**
701 - Establish a lead agent for Education, Research, and Quality Improvement				
	701.1 - Designate a lead agent for Education			
	701.2 - Designate a lead agent for Research			
	701.3 - Designate a lead agent for Quality Improvement			
	701.4 - Empower lead agents to work within system and with system leadership			
702 - Establish Education lead agency				
	702.1 - Establish a plan for ongoing education for physicians, nurses, and other providers			
	702.2 - Establish standards of education for each level of provider			
	702.3 - Establish method of assessing compliance at each clinical level			
	702.4 - Each trauma center has lead agent and plan for education			
703 - Establish Research lead agency				
	703.1 - Establish a plan for research for the system and for each center			
	703.2 - Develop and implement an injury database			
	703.3 - Ensure each trauma center collects data for all trauma patients			
	703.4 - Ensure each center contributes data to the system database on an established schedule (ie monthly, bimonthly, etc)			
	703.5 - Ensure Research agency also collects and analyzes population-based data in conjunction with efforts of Prevention Agency			
	703.6 - Agency analyzes data from system on a routine basis to assess for gaps in care and areas of potential improvement			
	703.7 - Agency informs system leadership regarding gaps in care based on injury data and population data and makes recommendations for system and trauma center improvement based on this data			
704 - Establish lead agency for QI				
	704.1 - Each trauma center establishes lead agent/agency for QI			
	704.2 - System establishes overall lead agent/agency with authority to direct hospital QI and system QI			
	704.3 - QI agents work with Prevention agents and Research agents in order to identify gaps in care based on collected data			
	704.4 - QI agents work with Prevention agents and Research agents in order to develop interventions for improved care within centers and the trauma system based on identified gaps			
